# Under cadmium stress, silicon has a defensive effect on the morphology, physiology, and anatomy of pea (*Pisum sativum* L.) plants

**DOI:** 10.3389/fpls.2022.997475

**Published:** 2022-10-06

**Authors:** Samira A. F. El-Okkiah, Amira M. El-Tahan, Omar M. Ibrahim, Mohamed A. Taha, Shereen Magdy Korany, Emad A. Alsherif, Hamada AbdElgawad, Esmaeel Z. F. Abo Sen, Mohamed A. Sharaf-Eldin

**Affiliations:** ^1^ Deparment of Agriculture Botany, Faculty of Agriculture, Kafrelsheikh University, Kafr Elsheikh, Egypt; ^2^ Plant Production Department, Arid Lands Cultivation Research Institute, The City of Scientific Research and Technological Applications (SRTA)-City, Alexandria, Egypt; ^3^ Department of Horticulture, Faculty of Agriculture, Minufiya University, Minufiya, Egypt; ^4^ Department of Biology, College of Science, Princess Nourah bint Abdulrahman University, Riyadh, Saudi Arabia; ^5^ Biology Department, College of Science and Arts at Khulis, University of Jeddah, Jeddah, Saudi Arabia; ^6^ Department of Botany and Microbiology, Faculty of Science, Beni-Suef University, Beni Suef, Egypt; ^7^ Cotton Research Institute, Agricultural Research Center, Giza, Egypt; ^8^ Department of Horticulture, Faculty of Agriculture, Kafrelsheikh University, Kafr Elsheikh, Egypt

**Keywords:** cadmium, Pea, oxidative stress, silicon, MDA

## Abstract

Soil pollution with cadmium (Cd) is a serious threat to plant growth and development. On the other hand, silicon (Si) can support plants to cope with Cd stress. However, the Cd stress mitigating impact of Si reduction in pea (*Pisum sativum* L.) is not known. The objective of this study is to see if and how Si can reduce Cd toxicity. To the end, a greenhouse pot experiment was performed twice during the 2018/2019 and 2019/2020 seasons to investigate the effect of Si on the growth, anatomy, and biochemistry of Cd stressed peas plants. Cd exposure increased the contents of Cd ions in the root and shoot of pea plants. Consequentially, Cd accumulation in pea tissue significantly reduced plant growth i.e., plant height, leaf area, and shoot and root dry weights. The effect of Cd was concentration-dependent, where at low concentration (50 mg/kg soil), the plant height was 94.33 and 97.33cm and at high concentration (100 mg/kg soil), it was 89.0 and 91.0 cm in the two seasons, respectively. This growth reduction can be explained by the decrease in plants’ photosynthesis, whereas plants exposed to Cd toxicity had lower chlorophyll levels. At the anatomy level, high Cd concentrations resulted in anatomical abnormalities such as an unusual vascular system, abnormal lignification in the pith parenchyma, and enlarged cortical cells. Moreover, all Cd concentrations resulted in a highly significant decrease in stomatal area and stomatal density (the number of stomata per mm^2^). In addition to growth inhibition, Cd-induced oxidative damage to pea plants as indicated by increased hydrogen peroxide (H_2_O_2_) and Malondialdehyde (MDA) levels. To reduce stress toxicity, plants treated with Cd at 50 and 100 (mg/kg) showed a significant increase in antioxidant capacity. Peroxidase (POD) enzyme activity was significantly increased by 41.26%, 28.64%, 77.05%, and 60.77% in both seasons, respectively. Si at 300 ppm under Cd (100 mg/kg) stress conductions considerably reduced (MDA) contents by 29.02% and 29.12%, in the two seasons, respectively. The findings pointed out that Si’s ability to protect pea against the oxidative stress caused by Cd toxicity.

## Introduction

*Pisum sativum* L. (pea) is an annual plant that grows in the winter, and it is one of the most important energetic crops in the Leguminosae family (Fabaceae). The immature seeds of this crop are eaten as fresh, frozen, or canned vegetables. Pea, starchy vegetable with high protein, has long been regarded as a low-cost, easily available source of protein due to their high levels of key amino acids such as tryptophan and lysine (Orzoł et al., 2022). Carbohydrates (fiber and starch) are also the primary components of peas, accounting for 20 and 46% of dry matter in seeds. Pea seeds are also rich in vitamins (A, B6, C, and K), minerals (P, Mg, Cu, Fe, and Zn) and lutein content (Eid et al., 2019). Pea seeds are extensively farmed including Egypt’s Nile Delta. In Egypt, the total area planted with pea was 4326.9 (ha), with a total yield of 14 t/ha of green legumes (FAO, 2014). However, different sorts of soil contaminants [including heavy metals mercury (Hg), lead (Pb), and cadmium (Cd)] have an impact on vegetable growth of the West Nile Delta soil (Khalifa and Gad, 2018). Therefore, the development of agriculture in a low-input and sustainable way is urgently needed to maintain pea growth under heavy metal contaminations.

Plants are naturally susceptible to various stress factors, including abiotic and biotic stressors. In this context, excess irrigation from drawing wells, agricultural chemical fertilizers, and quick industrial expansion has increased the number of toxic metals in agricultural soils that negatively impact the soil-plant environment system (Gadallah and Sayed, 2014; Jali et al., 2016). For instance, heavy metals-contaminated water for crop irrigation survived cadmium-rich rocks, smelting, mining, sewage sludge application, or excessive use of phosphate fertilizers (Hooda et al., 2007; Kabta-Pendias, 2011; Alloway, 2013). Because heavy metal stress negatively impacts crop development and productivity, it is of great interest to researchers (Gill, 2014). In recent years, there has been a lot of focus on heavy metals and trace elements in the environment, specifically in soil and water, as well as their effect on plant nutrition and productivity. (Gaafar et al., 2012) who reported that carbohydrate content in white radish and Triticum aestivum L. significant decline as the heavy metal (cadmium and lead) concentration increased. For example, using polluted soil or water for agricultural cultivation reduces productivity and produces contaminated food grains and vegetables, both of which are harmful to human health (Galal and Shehata, 2015a). (Ayangbenro and Babalola, 2017) and (Zhang et al., 2018) also found that trace metals (TM) influence public health and the ecological system. Crops and vegetables that grow in heavy metal-polluted soil accumulated a higher heavy metal concentration. Using this contaminated land for crop cultivation has a negative impact on plant cultivations, leading to lower production efficiency and contaminated food grains and vegetables, both of which are harmful to plant and human health (Singh and Agrawal 2010). Because of its water solubility and movement within the ecosystem, Cd induces necrosis and chlorophyll degradation and alters nutrient absorption, cellular metabolism, carbon fixation, and membrane function (Ahmad et al., 2015; Jali et al., 2016). Cd stimulates the production of ROS, which causes severe damage to various cell organelles and, as a result, inhibits plant growth (Ahmad et al., 2016). Moreover, Cd increases the antioxidant defense systems of stressed plants as a defense strategy to cope with induced oxidative stress (Peng et al., 2017). Furthermore, Cd stress can alter gene expression in a variety of ways (Farooq et al., 2016). Thus the phytotoxic impact of such compounds on the growth, development, and productivity of economical crops should be deeply investigated.

Silicon (Si), is not an essential element for higher plant growth, but it has improved the growth of several crops including rice, wheat, barley, and cucumber. Thus, Si is widely used as a fertilizer in numerous nations to boost productivity and ensure long-term yield (Ma et al., 2015). It has been shown to mitigate abiotic stress (e.g., salt stress, metal toxicity, drought stress, radiation damage, high temperature, and freezing), biotic stress (pests and fungi and bacteria infections), and nutritional imbalance (Ma and Yamaji, 2006). Particularly, multiple studies have recently demonstrated that Si represents a significant alternative to provide tolerance against the negative effects of various metals such as Cd^2+^ (Shi et al., 2017) and Ni^2+^ (Abd-Allah et al., 2019). Nonetheless, the role of Si in sustaining plant growth in the presence of toxicants remains largely unknown. According to (Rizwan et al., 2016), Si-mediated Cd toxicity mitigation includes external and internal mechanisms, such as increased nutrient uptake and decreased metal uptake and translocation. Furthermore, the formation of Cd–Si co-complexation in the cell wall reduces Cd transport to plant tissues (Ma et al., 2015). Again, (Ribera-Fonseca et al., 2018) demonstrated that reduced heavy metal uptake by Si could be attributed to root exudation of secondary metabolites such as phenolic compounds and organic acids. Secretions of these compounds chelate with metal cations and limit their entry into plant roots. Furthermore, Si’s protective effect against heavy metals may be related to the activation of enzymatic antioxidant processes (Farooq et al., 2016; Abd-Allah et al., 2019). According to (Hussain et al., 2015), Si application increased CAT and GPx activity in Cd-treated wheat plants compared to control plants. In this context, (Shi et al., 2017) found that increasing antioxidant activities, particularly those involved in H_2_O_2_ eradication, can reduce ROS generation in crops subjected to biotic stress Our study’s objectives were to (1) determine how Cd toxicity affects pea growth, anatomical features, and biochemistry (antioxidant enzyme activities and non-enzymatic antioxidants) of Pea plants, which is one of the most important crops in Egypt, (2) gain insight into the possible mechanisms involved in Si-mediated Cd detoxification. We investigated the hypothesis that less Cd accumulation and changes improved physiological and antioxidant defense systems underlie the Cd stress mitigating effect of Si treatment.

## Materials and methods

### Plant material, growth conditions, and treatment techniques

Pisum sativum L. cv. Master-B seeds were obtained from the Ministry of Agriculture in Egypt. Pot experiments were carried out in the greenhouse of the Department of Agricultural Botany, Faculty of Agriculture, Kafr El-Sheikh University, Egypt, during the two successive seasons of 2018/2019 and 2019/2020. Plastic pots (30 cm in diameter) were filled with 10 kg of clay loam soil (**Table 1**). After five minutes of seed surface sterilization with 2% sodium hypochlorite, the seeds were carefully washed and rinsed multiple times with sterile water. Before planting, the soil used in this experiment was fertilized with calcium super phosphate fertilizer (P_2_O_5_ 15.5%) that was added at the rate of 5 g/kg. Six healthy seeds were planted in each pot on the 19^th^ of September 2018/2019 and on the 17^th^ of September 2019/2020 seasons respectively. Under ideal conditions, in aerated full-nutrient media in plastic pots (Sandalio et al., 2001). The treatments were: control soil or Cadmium (CdCl_2_) treated soil at low concentration (50 mg/kg soil) and high concentration (100 mg/kg soil). Then silicon or Si(OH)_4_ (Sigma Chemical Company, St. Louis, MO, USA). Si treatments were introduced when seedlings were 25 days old at three concentrations of 100, 200, and 300 ppm Si. Plant foliar application was carried out two times using a one-hand pressure sprayer; the first application was 25 days after planting and the second after 45 days from sowing (at vegetative and flowering stages, respectively). The application was in the early morning when the stomata were open, allowing for better foliar penetration. Tween-20 was added at 0.1% (v/v) to applications as a surfactant to ensure optimal penetration into leaf tissues. The pots were then distributed into 12 treatments/groups, each treatment of 3 pots. A split-split plot design was used in this experiment with three replications.

**Table 1:** Soil physical properties.

Properties/Seasons		Fine sand %				Silt %		Clay %			Texture class
2018/2019		8.99				37.8		56.4			Clay
2019/2020		9.79				38		52.1			Clay
2-Soil chemical properties											
Properties/		PH		E.C		Cations (mg/L)				Anions(mg/L)	
Seasons				ds/m		Ca^++^ Mg ^++^ Na ^+^ K^+^				CO_3_ H CO_3_ Cl SO_4_	
2018/2019		7.1		1.44		105.6 86.8 70.0 4.03				-- 7.5 90.0 196.93 -- 8.8 90.31 198.1	
2019/2020		7.5		1.51		104.4 87.91 69.49 5.38					
3-Soil macro, micro and heavy metals nutrients available											
Elements /Seasons	Micro nutrients (ppm)				Heavy metals (ppm)		Macro nutrients (mg/L)				
2018/2019	Zn		Fe		Cd	Pb	N	NO_3_	NH_4_	P	K
2019/2020	85		14.93		0.12	18	2300	157	53	21.4	219.4
	89		15.35		0.37	19.5	2500	125	46	28.5	302.2

### Morphological growth and yield parameters

#### Morphological growth parameters

The roots and shoots of control plants, Cd, and/or Si-treated plants were harvested at the early flowering stage. The vegetative growth indicators such as plant height (which was measured from the soil surface to the top of the main stem), as well as shoot and root dry weights, were measured. Shoots were removed from the plant organs and oven-dried at 70°C until a constant weight was attained as (g) per plant, leaf area (cm^2^) as measured with a leaf area meter LI-3100 Area Meter).

#### Yield and yield components characteristics

At harvest, 80 days after sowing, the number of pods per plant and the number of seeds per plant were counted. Total seed yield per plant (g) by gathering all seed weight per plant at the moisture content of 10-12%, weights of 100 seeds were determined.

#### Physiological and biochemical analysis

Some physiological and biochemical parameters were assessed during the early flowering stage as follows:

#### Determination of Cd level

Cd was determined using an atomic absorption spectrophotometer in the digest acid solution of the root and shoot samples, according to (Chapman and Pratt, 1982.)

#### Concentration of chlorophyll pigments

The photosynthetic pigments (chlorophyll a, b, and total chlorophyll) were determined using the fourth leaf from the tip of the pea plant after 50 days from sowing during both seasons. The concentrations of chlorophyll (Chl.) were calculated as g/0.5 g fresh weight from the leaves. The pigments were extracted with 5 mL of N-N Dimethyl formamide before being stored in the refrigerator for 24 hours in the dark. The absorbency of the samples was measured using a spectrophotometer at 664 and 647 nm wavelengths. The concentration pigments was determined according to Moran, 1982.

#### Protein and total carbohydrates concentration in the seeds

The total nitrogen content of pea seeds was determined using the micro-Kjeldahl method, as described by (A.O.A.C., 1995). The proportion of protein in the seeds was computed by multiplying the total N % by 6.25 to get the percentage of protein in the seeds. Total soluble carbohydrates (TSC) were determined according to (Yemm and Willis, 1954).

#### Oxidative stress markers

The contents of malondialdehyde (MDA) and H_2_O_2_ were measured. A plant tissue sample (shoot) of 0.2 g was grounded in 5 ml of 0.1% TCA and centrifuged at 10,000g for 5 minutes. To 1 ml of the supernatant aliquot, 4 ml of 20% TCA containing 0.5% thiobarbituric acid (TBA) was added. The concentration of MDA (mol g 1 FW) was measured using (= 155 mM 1 cm1).

H_2_O_2_ levels were determined by grounding 0.5 g of fresh plant tissues (root) with 5 ml of trichloroacetic acid (TCA 0.1%) and centrifuging at 12,000g for 15 minutes at 4°C. A total of 0.5 ml of supernatant was mixed with 0.5 ml of 10 mM KPO4 buffer (pH = 7.0). Absorbance was measured at 390 nm using spectrophotometer (Velikova et al., 2000).

#### Antioxidant enzyme activity testing

pt?>At 4°C, pulverized shoot samples (100 mg) were blended in 1 mL of 0.2 M potassium phosphate buffer (pH 7.0) containing 0.1 mM EDTA to estimate antioxidant enzymatic tests. The filtrates were utilized to assess the antioxidant enzymes (catalase (CAT) and peroxidase (POD) activities after centrifugation at 15 000 g for 20 minutes. Measuring enzyme activity methods were according to Rios-Gonzalez et al. (2002). CAT activity was measured spectrophotometrically by looking for a decrease in H_2_O_2_ absorbance at 240 nm. The potassium phosphate reaction buffer was 50 mM. A phosphate buffer and 15 mM H_2_O_2_ were used (pH 7.0). One minute after the reaction began, the absorbance change was measured (e = 43.6 mM1 cm1). The guaiacol substrate was used to determine POD activity. The greatest absorption of the tetraguaiacol produced in the reaction was at 470 nm. In a 50 mM phosphate buffer (pH 7.0) solution with 0.3% H_2_O_2_ and guaiacol, the enzyme was evaluated (1%).

### Anatomical studies

A minimum of 5 samples of pea plant stem were randomly collected after 30 and 50 days from sowing (specimens 1cm long were taken from the fourth upper internode). For 48 hours, the sampled material was fixed in FAA (50% ethanol + 5% formaldehyde + 10% glacial acetic acid in water). Two washes in 70% ethyl alcohol were performed. Dehydration was achieved by passing the samples through a series of ethyl alcohol concentrations (75–100%). Each sample was passed through a mixture of xylol and absolute ethyl alcohol at the following percentages: 25%, 50%, 75%, and pure xylol in the final two changes for each dilution. Within 12 hours, the paraffin shavings reagent containing samples was saturated. To remove all traces of xylol, two changes of paraffin were performed. Samples were immersed in melted Paraffin in embedding paper trays, then quickly cooled in cold water. Rotary Microtome (Leica RM 2125 apparatus) sections (10-12 microns thick) were cut, and paraffin sections were fixed to the slides with Albumin. Slides were dried completely in a dry oven at 50°C for 24 hours. The slides were first immersed in two changes of xylol for about 10 seconds before being transferred to a jar containing equal parts absolute ethyl alcohol and xylol for 5 minutes. The sections were immersed in a series of descending ethyl alcohol dilutions ranging from absolute to 5%. After that, the sections were stained for 10 minutes in a jar containing 1% safranin, and the excess stain was washed away. Sections were stained in a jar containing 1% light green for 1 minute, then cleared in xylol, mounted in Canda Balsam, and ready for microscopic examination (Ruzin, 1999). Each slide was photographed after five readings were examined with an electric microscope (Leica DM LS) and a digital camera (Leica DC300).

#### epi-fluorescence Teicnec

From the fourth upper internode, a series of hand sections of stems were prepared at 1 mm intervals. The free hand sections were stained with 001% Fluorol yellow 088 dissolved in lactic acid for 30 minutes and then washed in distilled water for suberin visualization in fluorescence microscopy (Lux et al., 2005). Prior to observation, the samples were placed in a drop of 1% FeCl_3_ dissolved in 50% glycerin. Toluidine blue was used to stain the sections for bright-field observations. The sections were photographed with an Olympus DP 72 digital camera and examined with a Zeiss Axioskop2 plus epifluorescence microscope.

#### H_2_O_2_ localization in pea roots

We looked at the primary and lateral roots of pea that had been exposed to Cd for seven days. The approach of (Thordal-Christensen et al., 2000) was modified for H_2_O_2_ localization. Fresh, hand-cut samples were incubated for 30 minutes at room temperature in a 1 mg/mL solution of 3,3′-DAB-HCl, pH 3.8 (Sigma). H_2_O_2_ production was represented by a reddish-brown hue. After washing the slides for 10 minutes, we fixed the DAB-stained tissues in 2.5% glutaraldehyde and Nikon microscope was used to examine the results.

#### Stomatal density and guard cell size

Leaf stomatal density was calculated (Radoglou and Jarvis, 1992). The leaf’s abaxial epidermis was cleaned with a degreased cotton ball before being carefully smeared with the nail varnish for about 20 minutes. The thin film was peeled off the leaf surface and under a photomicroscope system with computer attachment, the number of stomata (s) and epidermal cells for each film strip were counted.

### Soil properties

Before sowing pea seeds, soil samples were taken from the major root zone. The soil samples were airdried, crushed, and passed through a 2 mm sieve before being analyzed for physicochemical properties. According to (Bouyoucos, 1965), each sample was prepared to form soil texture and used the hydrometer method. pH and EC meters were used to measure soil pH and electrical conductivity (EC) in a 1:2 soil: water suspension (Hati et al., 2007). A calcimeter was used to measure calcium carbonate (CaCO_3_). Heavy metals (Pb and Cd) in soil were extracted and measured using an Atomic Absorption Spectrophotometer (Gasco and Lobo, 2007).

### Statistical analysis

Using SPSS for Windows, an analysis of variance (ANOVA) with *post hoc* Duncan (1955) Multiple comparison test was performed (Ver. 13.0, SPSS Inc., USA). The significance of the means among controls and treatments was estimated using the probability level of P > 0.05.

## Results

### Morphological growth, yield, and yield components parameters

#### Morphological growth parameters

The effects of Cd and or Si treatments on plant height, leaf area, shoot dry weight, and root dry weight were measured (**Table 2**) Cd reduced plant height, leaf area/plant, shoot dry weight, and root dry weight of pea plants. Increasing cadmium concentrations progressively reduced the average plant height, where it reached 94.33 and 97.33 cm, at the low concentration while at the high concentration, it reached 89.0 and 91.0 cm compared to controlled plants in both seasons. Similarly, the average leaf area was decreased (**Table 2**) and at high concentrations of Cd, the maximum reduction in leaf area was achieved. A significant decrease in the average values of both the dry weight of shoot and root compared to the control was recorded. Under high concentrations, the average weights of shoot dry weight reached 4.57 g, 5.65 g, 1.35 g, and 1.80 g for dry weight of root, respectively. Si treatment reduced Cd’s negative effects on growth parameters. At 300 ppm exogenous Si to Cd (100mg/k) treated plants increased plant height by 37.45% and 36.26%, leaf area by 33.99% and 31.87%, dry weight of shoot by 31.48% and 37.16, and root dry weight by 36.49% and 29.97%, in the two seasons respectively.

**Table 2:** Effect of foliar application of silicon on pea (plant height, leaf area, shoot and root dry weight) grown in different concentrations of cadmium (Cd) during 20182019 and 2019/2020 growing seasons.

Treatment (g)		Plant height (cm)		Leaf area/plant (cm^2^)		Shoot dry weight (g)		Root dry weight (g)	
Cd mg/kg	Si ppm	2018	2019	2018	2019	2018	2019	2018	2019
0	0	126.67 b	128.67 b	826.473b	870.243b	6.27 e	7.29 ef	1.72 de	2.28 def
	Si100	135.33 a	137.33a	845.573b	889.343b	10.33 c	11.35 c	3.78 c	3.34 c
	Si 200	138.00 a	140.00 a	878.350a	922.120a	12.10 b	13.13 b	4.22bc	4.44 b
	Si 300	138.67a	140.67a	892.257a	936.027a	13.77 a	14.81a	5.55 a	5.44 a
50 mg/kg	0	94.33 f	97.33 e	648.687g	704.007g	5.07 fg	6.11 gh	0.52 g	0.97 g
	Si100	121.00 cd	124.0bc	707.333ef	762.653ef	6.40 e	7.44 e	1.85 de	2.30de
	Si 200	124.67 c	125.67bc	741.733d	797.053d	6.83 e	7.89 e	2.28 d	2.73 cd
	Si 300	128.00 b	129.00 b	785.510c	840.830c	9.00 d	10.08 d	4.45 b	4.90 ab
10**0** mg/kg	0	89.00 f	91.00 e	537.910h	573.79 h	4.57 g	5.65 h	1.35 ef	1.80 ef
	Si100	112.33 e	114.33 d	629.853g	665.73 g	5.60 f	6.68 fg	1.05 fg	1.50 fg
	Si 200	117.00 de	119.00 cd	690.123f	726.00 f	6.47 e	7.55e	1.92de	2.37de
	Si 300	122bcd	124.00 bc	720.780e	756.66 e	6.67 e	7.75 a	2.12d	2.57 cde

Means that do not share a letter are significantly different.

#### Yield and yield components characteristics

Cd treatment resulted in a significant decline in seed yield (**Table 3**). Reduced number of pods per plant, fewer seeds per pod, and lower seed weight were among the factors shown to be responsible for lower seed yield in Cd-treated plants in this study. With increasing Cd concentrations, the number of pods and weight of 100 seeds declined progressively. The foliar spray of Si improved yield and yield components characteristics in Cd-stressed plants in both seasons.

**Table 3:** Effect of foliar application of silicon on pea (yield and yield components characteristics) grown in different concentrations of cadmium (Cd) during 2018/2019 and 2019/2020 growing seasons.

Treatment		Number of pods/plant		Number of seeds/pods		100 seed weight(g)		Seed yield plant^-1^(g)	
Cd mg/kg	*Si ppm*	2018	2019	2018	2019	2018	2019	2018	2019
0	0	11.33 cd	12.33 cd	6.17c	7.40 c	58.53 g	59.82g	29.26f	30.49g
	Si100	12.33 bc	13.33 bc	6.67bc	7.90 bc	69.63c	70.92c	34.81b	36.04bc
	Si 200	13.67 b	14.67 b	7.34b	8.57 b	73.44b	74.73b	36.72a	37.95b
	Si 300	15.33 a	16.33 a	8.17a	9.40a	77.51 a	78.80a	38.76a	39.99a
50 mg/kg	0	8.67 ef	9.67 ef	4.84e	6.07 e	48.77i	50.09 i	24.39 i	25.62i
	Si100	10.00 df	11.00 de	5.50de	6.73de	60.74f	62.06 f	30.37e	31.60i
	Si 200	10.33 d	11.33d	5.67d	6.90 d	61.74 e	63.06 e	30.87d	32.10dc
	Si 300	11.00 cd	12.00 cd	6.00cd	7.23 cd	63.79 d	65.11d	31.90cd	33.13d
1**00** mg/kg	0	8.33 f	9.33f	4.67f	5.90 f	45.88 j	47.20 j	22.94j	24.17ij
	Si100	8.67 ef	9.67 ef	4.84 ef	6.07 ef	46.07j	47.39 j	23.03j	24.26ij
	Si 200	10.33 d	11.33 d	5.67de	6.90 df	48.40 i	49.72i	24.20i	25.43i
	Si 300	11.33 cd	12.33 cd	6.17c	7.40c	51.37h	52.69 h	25.68h	26.91h

Means that do not share a letter are significantly different.

#### Biochemical analysis

##### Chlorophyll pigments levels

Data in **Table 4** illustrates that the content of chlorophylls (a and b) steadily decreased as Cd concentrations increased. The Si foliar treatment, on the other hand, may mitigate the deleterious effects of Cd imposed on chlorophyll content. Over a Cd dosage range of 50 to 100 (mg/kg), Si addition increased the levels of chlorophylls (a and b) in Cd-stressed plants.

**Table 4:** Effect of foliar application of silicon on pea (chlorophyll pigments) grown in different concentrations of cadmium (Cd) during 2018/2019 and 2019/2020 growing seasons.

Treatment		Chl. A (mg/g)		Chl. B (mg/g)		Total Chl. (mg/g)	
		F.W.		F.W.		F.W.	
Cd mg/kg	Si ppm	2018	2019	2018	2019	2018	2019
0	0	9.79 cd	10.99 d	4.81 cd	6.01 cd	16.42 f	17.62 f
	Si100	11.75 b	12.95 b	5.29 bc	6.49 bc	15.53 f	16.73f
	Si 200	10.39 bc	11.59 bc	5.67 ab	6.87 ab	18.39 de	19.59 df
	Si 300	13.61a	14.81a	6.28 a	7.48 a	19.42 c	20.62c
50 mg/kg	0	8.65 d	9.85 d	3.96 c	5.16 e	11.74 g	12.94 g
	Si100	13.51 a	14.71 a	6.42 a	7.62 a	20.62 b	21.82b
	Si 200	14.51 a	15.71 a	5.69 ab	6.89 bc	21.65 a	22.85a
	Si 300	14.50 a	15.70 a	5.31 bc	6.51 bc	21.51 ab	22.71 ab
1**00** mg/kg	0	8.91d	10.11 d	3.15 f	4.35 f	10.57 h	11.77h
	Si100	10.92 bc	12.12 bc	4.64 cdf	5.84 cde	18.59 cd	19.79cd
	Si 200	10.50 bc	11.70bc	4.36 de	5.56 de	17.59 c	18.79 e
	Si 300	13.32a	14.52 a	5.29 bc	6.49 bc	21.35 ab	22.55 ab

Means that do not share a letter are significantly different.

##### Chemical composition of plant organs (root, shoot, and seeds)

**Table 5** shows that Cd accumulation in the root was much greater than in the shoot. The rise in Cd concentration was given intravenously in all plant parts, including the shoot and root. With increasing cadmium concentrations, the level of proteins in pea seeds decreased significantly. Total protein content was also found to be significantly higher in pea plants treated with Si under control conditions. In contrast, Si treatments resulted in a significant increase under Cd stress conditions when compared to the control.

**Table 5:** Effect of foliar application of silicon on pea (Chemical composition of plant organs) grown in different concentrations of cadmium (Cd) during 2018/2019 and 2019/2020 growing seasons.

Treatment		Root Cd (µg g-1 D.W.)		Shoot Cd (µg g-1 D.W.)		Total carbohydrates (mg/g) D.W.		Protein (%)	
Cd mg/kg	Si ppm	2018	2019	2018	2019	2018	2019	2018	2019
0	0	1.48 i	2.06 h	0.65 g	0.78 g	42.33 cd	43.53 c	19.43 g	19.87 g
	Si100	1.41 i	1.99 h	0.53 g	0.66 g	41.47 d	41.92 d	20.07 fg	20.51 fg
	Si 200	1.35 i	1.93 h	0.49 g	0.62 g	45.57 a	46.77 a	22.43 d	22.87d
	Si 300	1.22 i	1.80 h	0.32 g	0.45 g	45.93 a	47.13 a	23.50 c	23.94 c
50 mg/kg	0	23.77b	24.02b	13.44 b	13.57 b	37.53 e	37.87 d	17.33 i	17.68 i
	Si100	16.73d	16.98d	11.33 c	11.46 c	41.47 d	41.81 c	22.40 d	22.75 d
	Si 200	12.48g	12.73 f	10.29 d	10.42 d	42.60 c	42.98 bc	24.58 b	24.93 b
	Si 300	10.54h	10.79 g	9.44 f	9.57 e	43.30 c	43.75 d	26.47 a	26.82a
100 mg/kg	0.00	36.70a	37.72 a	20.44 a	20.57 a	32.10 g	32.55 f	15.53 j	15.80 j
	Si100	18.81c	19.83 c	10.63 d	10.76 d	36.40 f	37.60 f	18.33 h	18.60 h
	Si 200	15.84e	16.86 d	9.66 f	9.79 e	42.40 cd	42.77cd	20.33 f	20.60 f
	Si 300	13.44f	14.46 e	8.55 f	8.68 f	44.31b	44.53 b	21.44 e	21.71 e

Means that do not share a letter are significantly different.

With increasing Cd concentrations, there is a significant decrease in total carbohydrate content in pea seedlings. The total carbohydrate content of pea shoots was significantly higher in treated plants with 200 or 300 ppm Si. However, when combined with 50mg of Cd, it had no significant effect.

##### Oxidative damage

**Figure 1:**
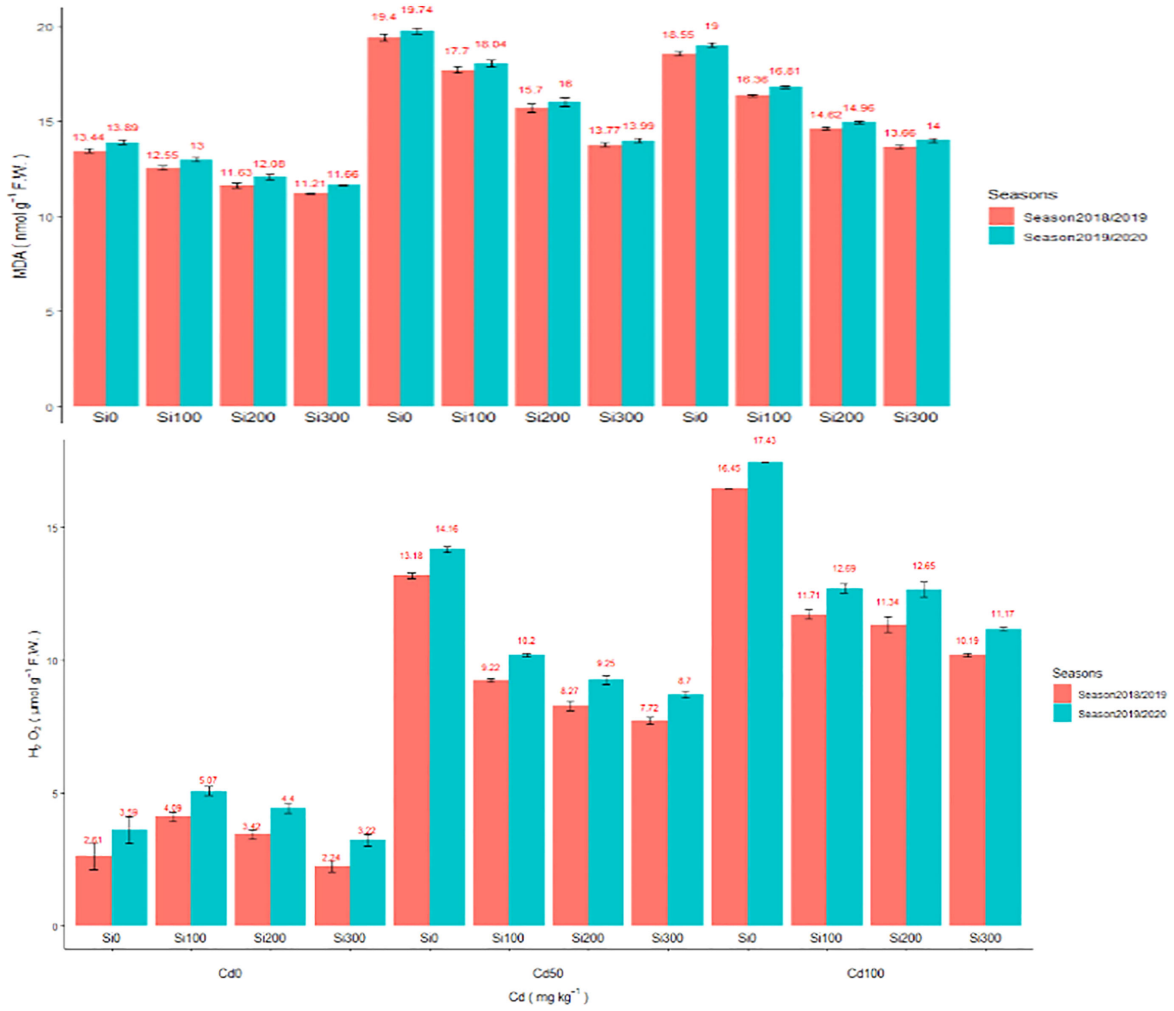
**(A)** Effect of foliar application of Silicon on pea MDA (nmol g-1 F.W.) grown in different concentrations of cadmium (Cd) during 2018/2019 and 2019/2020 growing seasons. **(B)** Effect of foliar application of Silicon on H_2_O_2_µmol g–1 FW grown in different concentrations of cadmium (Cd) during 2018/2019 and 2019/2020 growing seasons.

Cd at 50 and 100 (mg/kg) concentration dramatically increased the levels of ROS in pea leaves (**Figures 1A, B**). In comparison to the control, Cd concentrations of 50 and 100 (mg/kg) increased MDA by 38.20 and 44.34% for season one, 36.78%, and 42.11% for the second season, respectively. In the line with increased MDA level, the H_2_O_2_ level increased by 404.9% and 523.37% for the first season and 294.42% and 380.5% for the second season, respectively in the pea shoot. The use of Si dramatically reduced Cd toxicity by lowering ROS levels in pea shoot. Si at 300 ppm considerably reduced MDA contents by 29.02% and 29.12%, and H_2_O_2_ contents by 56.43% and 35.07% under in combination with Cd100(mg/kg) compared to Cd (100 mg/kg) treatment in the two seasons, respectively. In this study, under Cd stress, Si application at 300 ppm increased the activities of POD and CAT (**Figure 1**), and the increased antioxidant activated the defiance mechanism in plants, thus increasing the Cd resistance of pea plants.

##### Antioxidant enzyme activity testing

**Figure 2:**
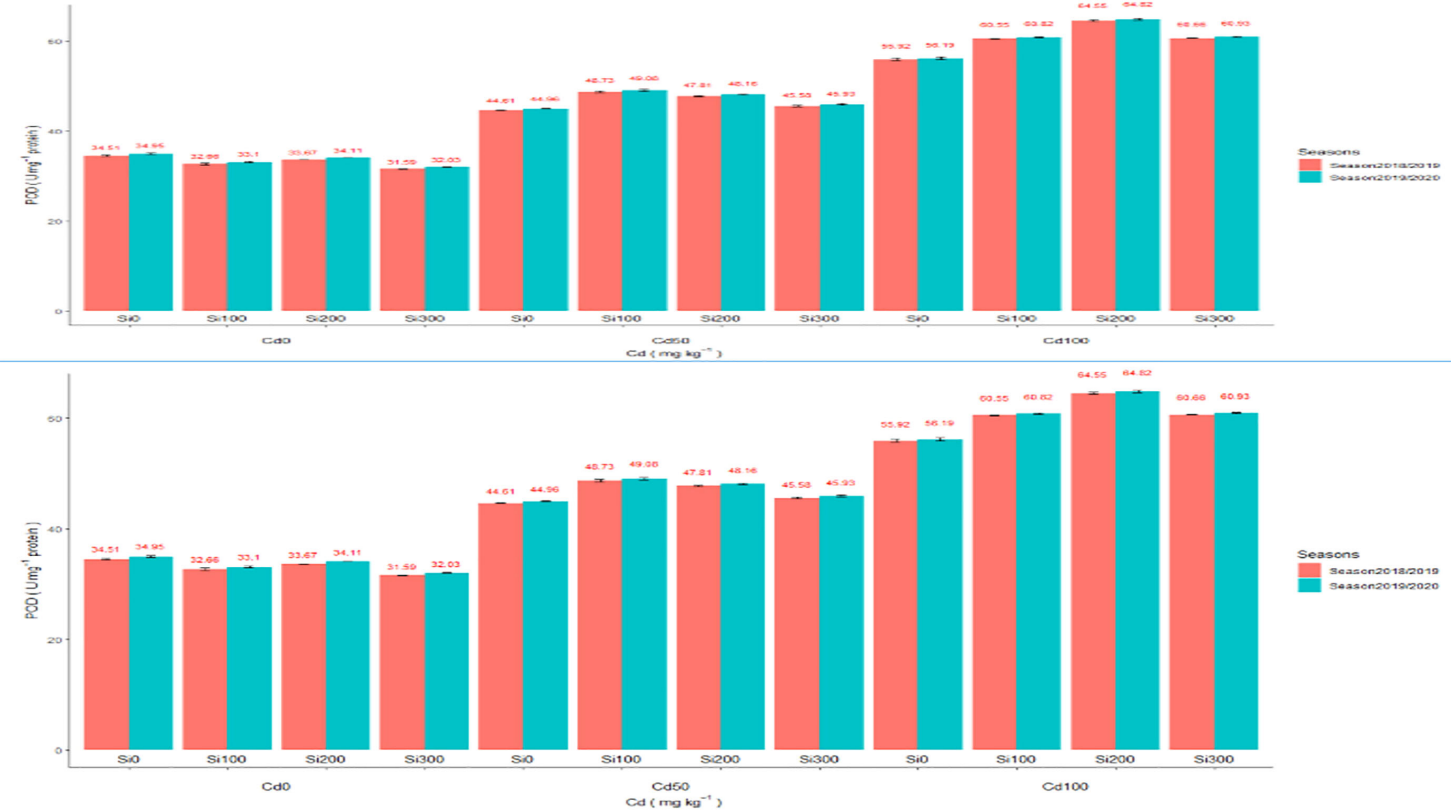
**(A)** Effect of foliar application of Silicon on pea (CAT U mg–1 protein) grown in different concentrations of cadmium (Cd) during 2018/2019 and 2019/2020 growing seasons. **(B)** Effect of foliar application of Silicon on pea POD U mg–1 protein grown in different concentrations of cadmium (Cd) during 2018/2019 and 2019/2020 growing seasons.

Assimilation of Cd elevated the enzymatic activity of oxidoreductase enzymes such as CAT and POD (**Figures 2A, B**). Compared to the control, Cd concentrations of 50 and 100 (mg/kg) significantly increased antioxidant capacity in the pea plants. In the shoot of pea plants, Cd at 50 and 100 mg/kg increased the activity of CAT enzyme by 70.09% and 186.82%, 62.43%, and 173.33% in both seasons, respectively. POD enzyme activity significantly increased by 41.26%, 28.64%, 77.05%, and 60.77% in the two seasons, respectively. Adding Si at a concentration of 300 ppm under Cd concentrations dramatically reduced Cd heights by increasing enzymatic antioxidant capacity in pea shoots.

### Anatomical studies

**Figure 3:**
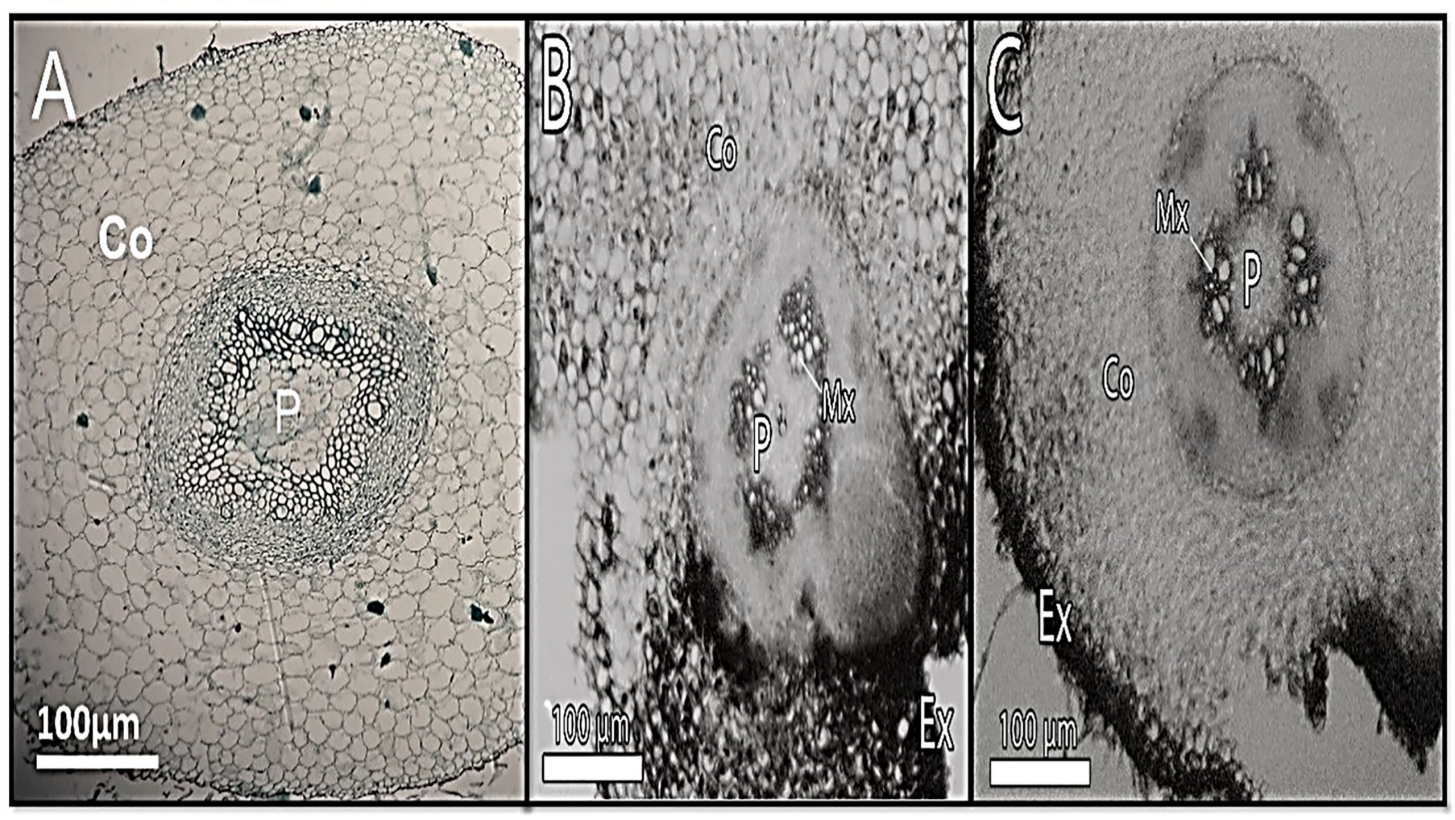
Show transverse section of a pea root. **(A)** - control, **(B)** - 100 mg/kg Cd-treated plant **(C)** - Cd100+Si 300 ppm displaying cortex (co)), pith (p), and meta xylem (Mx), localization of hydrogen peroxide (H2O2) (panel B).

**Figure 4:**
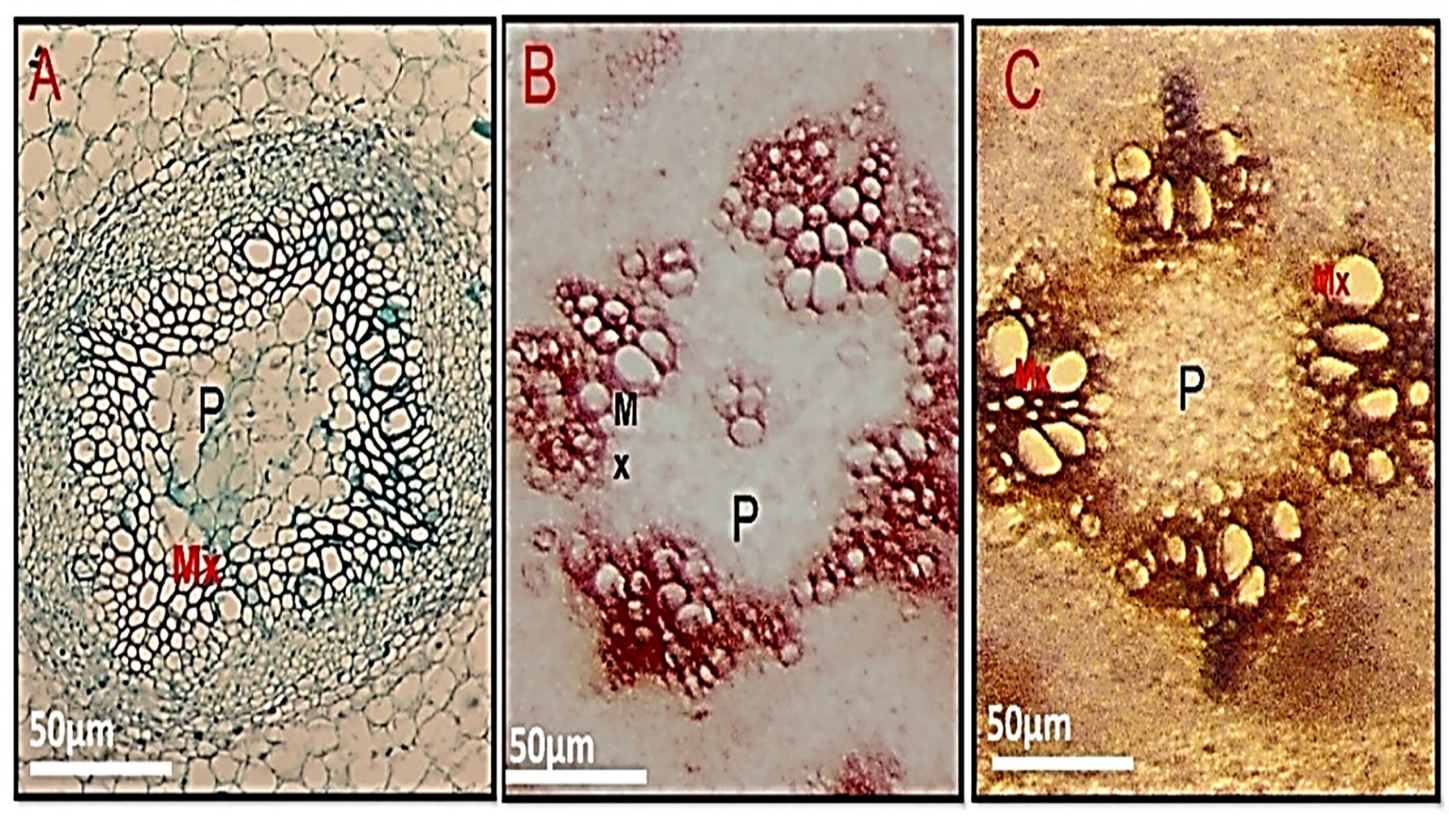
Shows a transverse section of a pea root. **(A)** - control, **(B)** - 100 mg/kg Cd-treated plant **(C)** - Cd100+Si 300 ppm pith (p), and metaxylem (Mx).

In Cd-treated plants, root diameter decreased when compared to control plants (**Figures 3**, **4** and **Table 6**). In control plant roots, 14 – 16 layers of the cortical cell were present, whereas, in Cd-treated plant roots, it was 7 – 10 layers (**Figures 3A, B**). In the more external region, refer to specific not exposed to Cd had uniseriate epidermis and hypoderm, whereas Cd increased two or three cell layers in the hypoderm, irregular and loosely arranged cortical cells (**Figures 3B**). Endodermal cells in treated roots were smaller and thicker walled than those in control plant roots (**Figures 4B**). A modified DAB-dependent method was used to examine the H_2_O_2_ in the roots of control and Cd-treated pea plants (**Figure 3B**). A distinctive reddish-brown coloration, indicating H_2_O_2_ occurrence, was found in root tissues. Furthermore, the H_2_O_2_ coloration in the Cd-treated roots’ cells and cell walls was noticeably stronger than in the control roots.

**Table 6:** Effect of foliar application of silicon on pea root cross-section grown in different concentrations of cadmium.

root cross-section	Treatments		
	Cd 0 mg/kg	Cd 100 mg/kg	Cd100+Si 300 ppm
Exodermis thickness µm	54.8 b	69.5 a	78.5 a
Number of rows cortex	14.55 a	9.55 c	12.55 b
Endodermis thickness (μm)	17.2 b	28.7 a	20.44 c
No. metaxylem	9.5 a	5.4 c	6.2 b
Area metaxylem elem. (μm)2	256.3 a	188.6 b	240.2 a
Pith thickness µm	170.0 a	88.67 b	156.7 a

Means that do not share a letter are significantly different.

**Figure 5:**
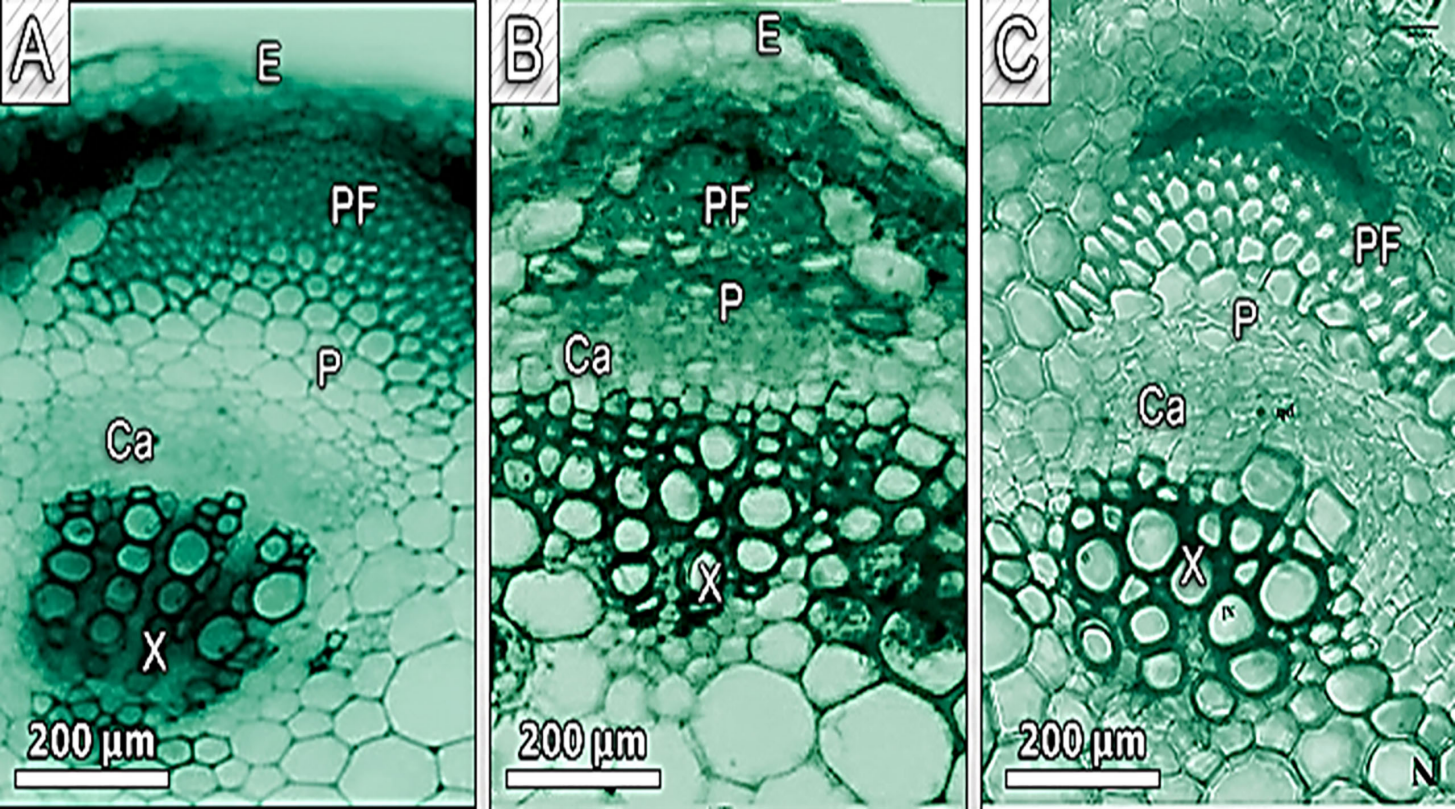
Show a transverse section of a pea stem. **(A)** - control, **(B)** - 100 mg/kg Cd-treated plant **(C)** - Cd100+Si 300 ppm pith (p), and meta vessel (E, epidermal; X, xylem; P, phloem; Ca, cambiumand;Pf, phloemfiber.

The diameter of the Cd-treated stem shrank (**Figure 5** and **Table 7**). The epidermal stem cells were thicker and the cortical cells in the Cd-treated stem occupied a smaller area (**Figure 5** and **Table 7**). The number of cortical layers in the experimental plant was 10-12, whereas it was 14 -16 in the control plant. The cambium ring of the Cd-treated stem was thin and the number of vessels was reduced in Cd-treated stems (Micrographs3-B). In comparison to control samples, stem xylem and phloem elements had a more subdued appearance. Phenolic compounds were discovered in higher concentration vessels of Cd-treated stem (**Figure 5B**).

**Table 7:** Effect of foliar application of silicon on pea stem cross-section grown in different concentrations of cadmium.

Stem cross-section	Treatment		
	Cd 0 mg/kg	Cd 100 mg/kg	Cd100+si300
stem diameter(µ)	3250.33a	2750.66b	3725.33 a
Thickens of epidermal cell (µ)	35.27c	60.33a	45.67b
Thickness of cortex layer (µ)	400.5 a	280.43 c	420.12 a
metaxylem vessels diameter (µ)	29 c	33b	37a
Area metaxylem elem. (μm)2	236.3 a	158.6 c	190.2 b
Pith thickness µm	190.0a	108.67c	146.7b

Means that do not share a letter are significantly different.

**Figure 6:**
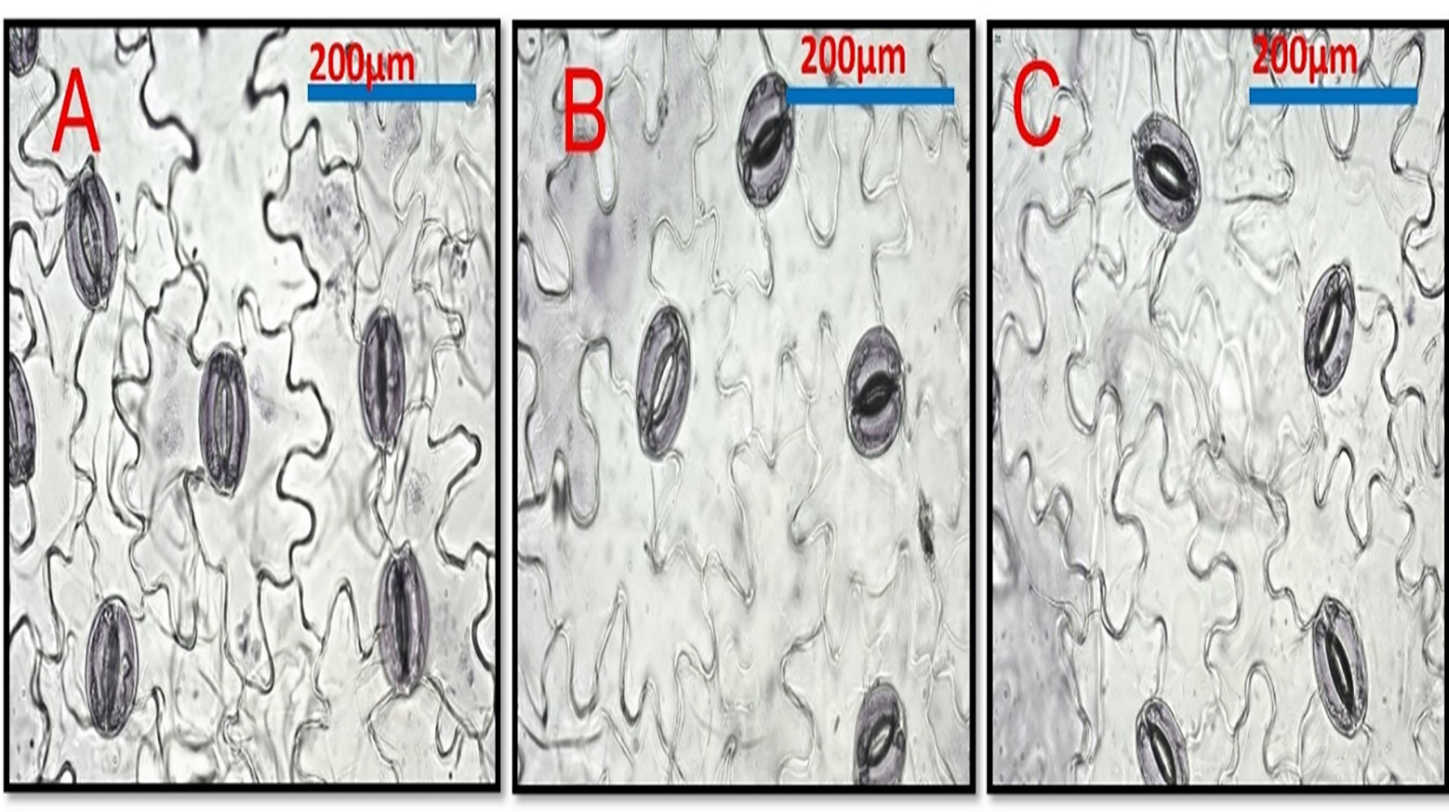
Shows the effect of Cd and Si on the stomata in pea leaf cells. Pea plants were grown with Cd 100 mg/kg. **(A)** Control leaf. **(B)** Pea leaf from Cd 100 mg/kg plants **(C)** Cd 100 mg/kg +Si 300ppm.

All Cd concentrations resulted in a highly significant decrease in stomatal area, and stomatal density (the number of stomata per mm^2^ leaf area) decreased as Cd concentration increased when compared to control plants (**Figures 6** and **Table 8**). Si treatment, in general, resulted in significant increases in the stomatal area when compared to Cd-treated plants.

**Table 8:** Effect of foliar application of silicon on number of stomata and size of stomata guard cell grown in different concentrations of cadmium.

*Stomatal aperture*	Treatments		
	Cd 0 mg/kg	Cd 100 mg/kg	Cd 100 mg/kg +Si 300ppm
Stomatal density (no·mm−2) Adaxial surface	26.88 a	11.5c	18.5 b
Stomatal length (μm) Adaxial surface	8.54a	5.88c	7.45b
Stomatal width (μm) Adaxial surface	5.2 a	3.7 c	4.44 b

**Figure 7:**
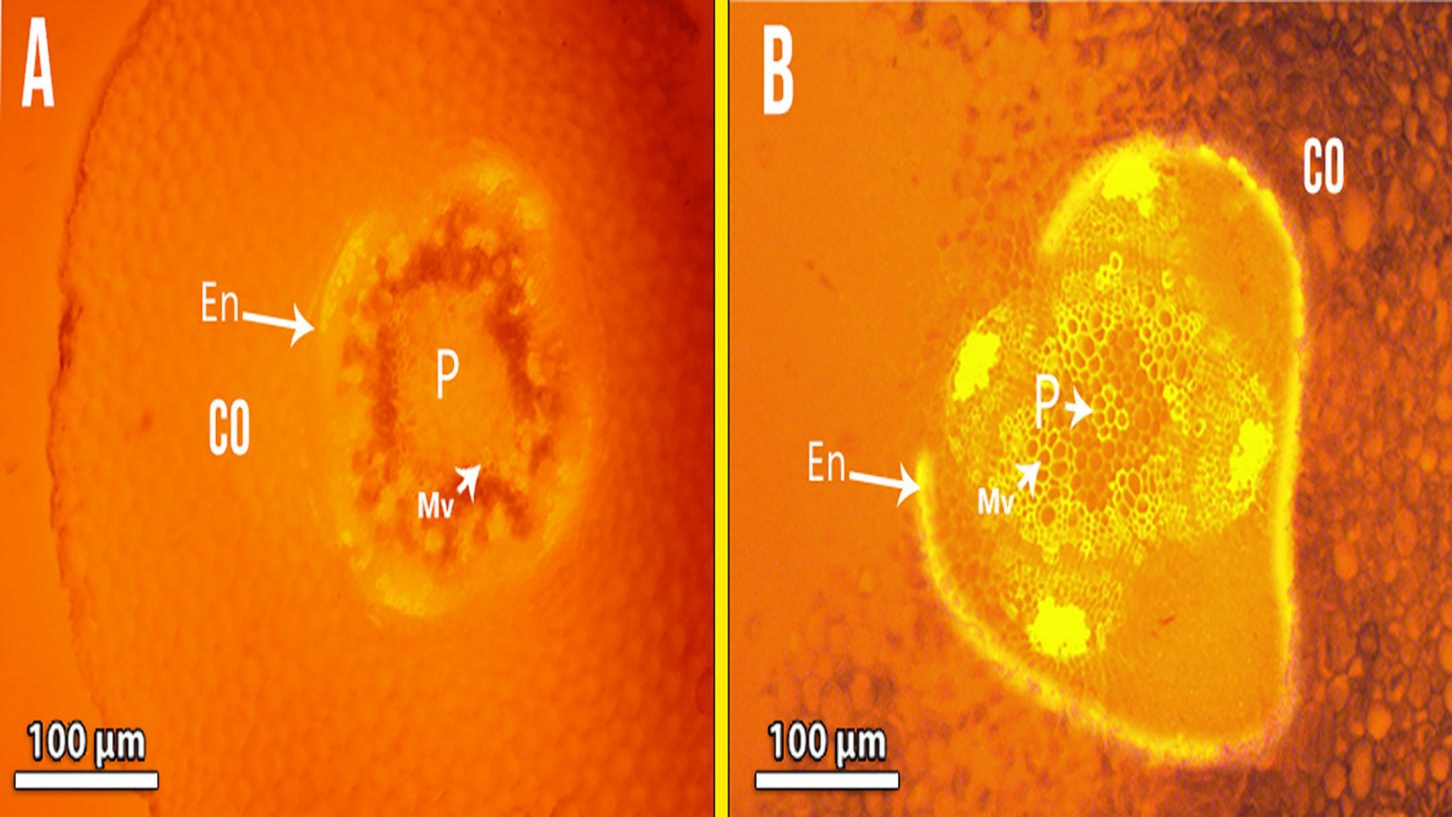
Showing Fluorescence photomicrography for lignin detection in the endodermis and parenchymatous pith in Cd-treated plant roots **(B)** compared with control **(A)** Co, cortex; En, endodermis; P, pith; Mv, meta xylem vessels.

Means that do not share a letter are significantly different.

(**Figure 6B**), compared to control plant roots (**Figures 6A**). Lignin detection in the endodermis and parenchymatous pith. Also. Lignin deposits in the xylem walls appeared yellow under brightfield microscopy (**Figures 6B**). Cd-treated plant roots had less parenchymatous pith. (**Figures 7**) the thicker endodermis is caused by a higher Cd concentration.

**Figure 8:**
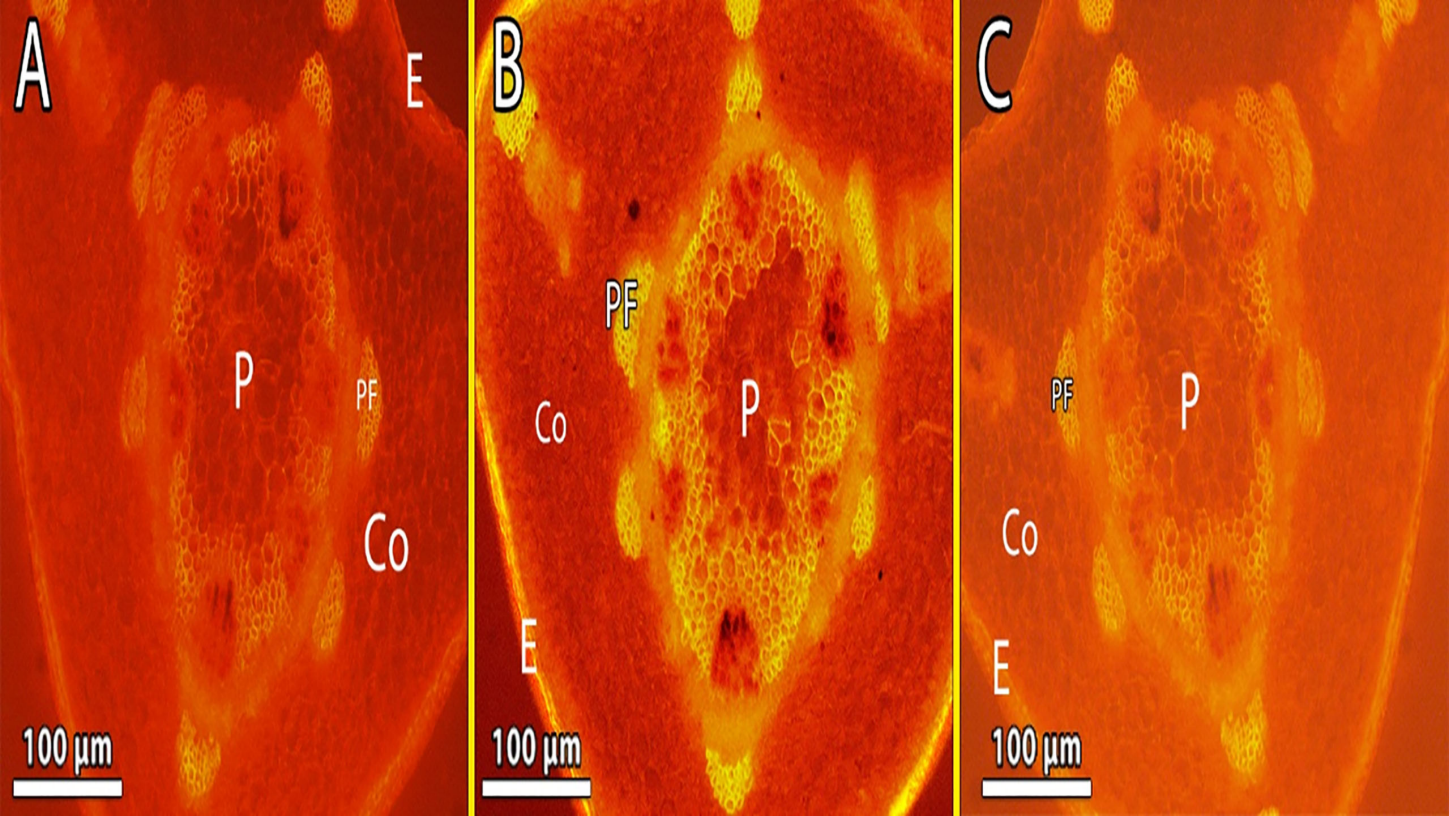
**(A–C)** Showing the lignin deposit in the stem epidermis cells was observed during the treatment, and Metaxylem development was found to be developed in the pith region exposed to the highest level of Cd **(B)** lignin deposits in the xylem walls appeared yellow under brightfield microscopy. E, epidermis; Co, cortex; P, pith; Ca, cambium and Pf, phloem fiber.

**Figure 8** show pea stems in normal conditions, where the epidermis’ outer wall has a thin cuticle layer. The epidermis has only one layer and the epidermal cells are tubular and small. Fluorescence findings indicate a thicker cuticle layer found at the outer wall of the epidermis. Pea stem responds to Cd stress by increasing lignification of endodermal cells and xylem vessels (**Figure 8B**). The highest concentration of Cd caused a lignin deposit in the stem epidermis cells. Cortical parenchyma of stem and pith of pea plants exposed to higher concentrations of Cd had thicker walls than the control. The pith parenchyma metaxylem development was found to be developed in the pith region (**Figure 8B**).

### Correlation analysis

**Figure 9:**
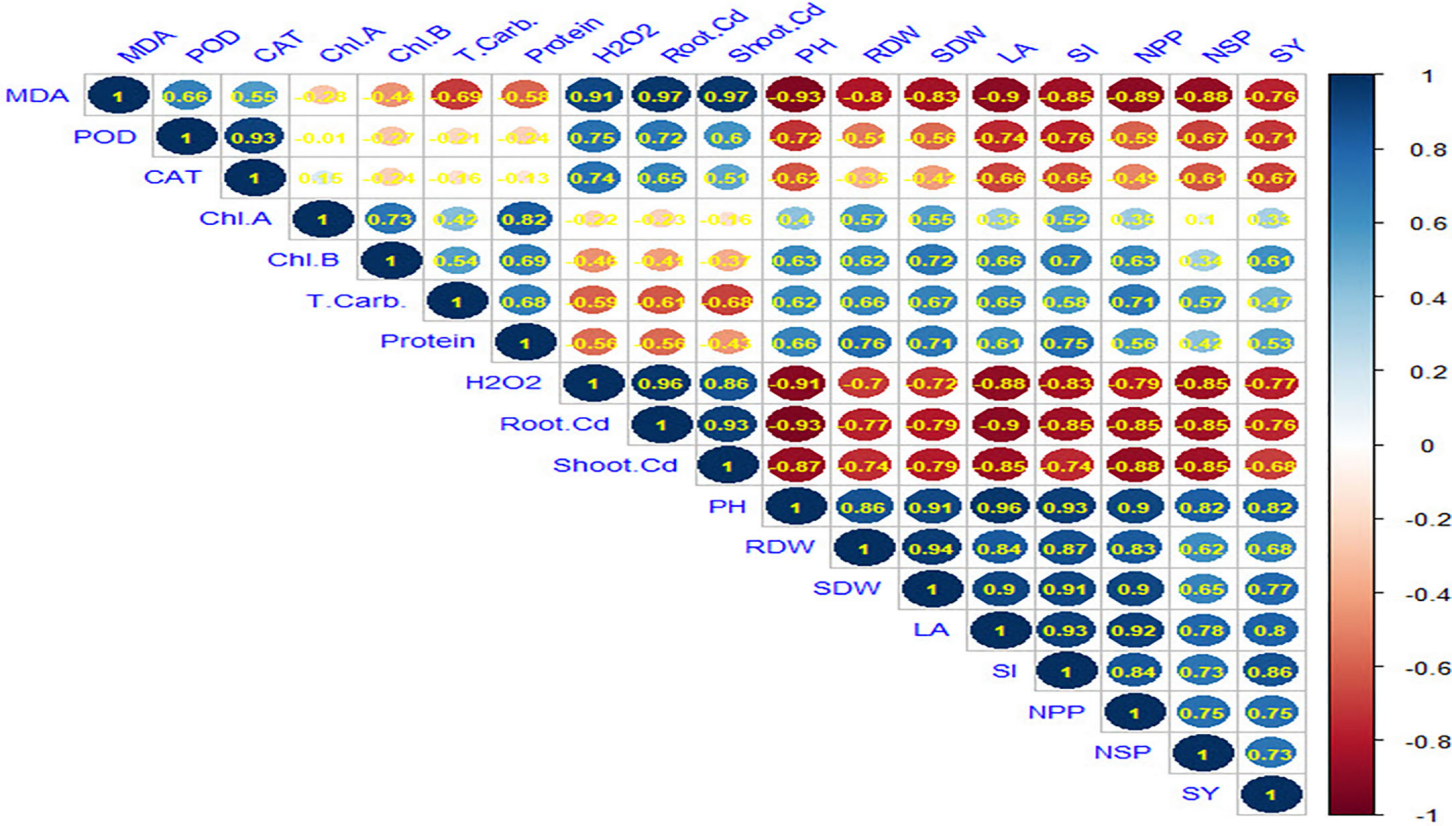
Spearman correlation matrix among malondialdehyde (MDA), antioxidant enzymes peroxidase (POD), antioxidant enzymes catalase (CAT), chlorophyll a (Chl.A), chlorophyll b (Chl.B), total carbohydrates (T.Carb.), Hydrogen peroxide **(**H_2_O_2_), root cadmium (Root Cd), shoot cadmium (Shoot Cd), plant height (PH), root dry weight (RDW), shoot dry weight (SDW), leaf area (LA), weight of 100 seeds (SI), number of pods/plant (NPP), number of seeds/pod (NSP), seed yield/plant (SY).

Pearson correlation matrix among the studied traits was performed and indicated by the color scale (**Figure 9**). Seed yield per plant (SY) was negatively and significantly correlated with MAD, POD, CAT, H_2_O_2_, root Cd, and shoot Cd. However, there was a weak correlation between SY and Chl a. On the other hand, SY was positively correlated with the agronomic characters (Ch. b, root dry weight (RDW), shoot dry weight (SDW), PH, LA, SI, NPP, and NSP) which were positively and significantly correlated with each other. Cadmium content in both root and shoot was positively and significantly correlated with MAD, POD, CAT, and H_2_O_2_.

**Figure 10:**
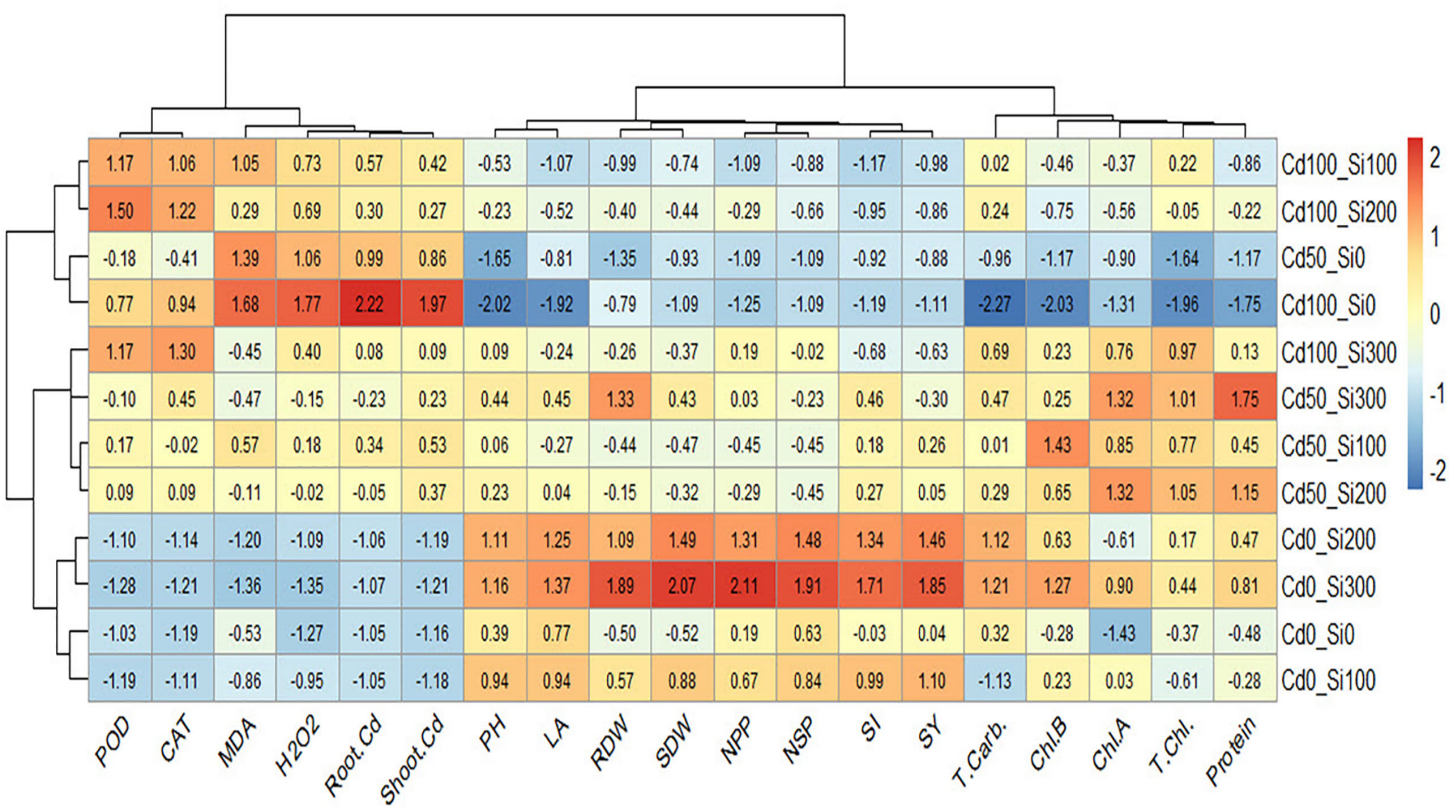
Heatmap showing the relationship between the studied traits and the treatments.

Heatmap presented a color scale comparison among treatments with respect to the studied traits based on scaled or standardized data (**Figure 10**). Cells with red color in the heatmap represent high values of the traits, while cells with blue color represent low values of the traits. Before constructing the heatmap, the data was standardized by subtracting the mean of each trait from every single value and dividing the result by the standard deviation of that trait. The standardization process is important to make the comparison feasible as the studied traits were measured in different measuring units. The results reveal that the treatment of Si at 300 under control conditions was the highest in seed yield (SY) represented by red color. The highest SY was positively associated with higher values (red color) of SI, NPP, NSP, LA, PH, SDW, and RDW. While it was negatively associated with lower values (blue color) of root Cd, shoot Cd, MAD, CAT, and POD. The highest values of root Cd, shoot Cd, MAD, and H_2_O_2_ occurred in the treatment Cd100_Si0 which own the lowest values (blue color) of PH, LA, Chl. a, Chl. b, total Chl., protein, and T. carbohydrates.

### Modeling the relationship between yield components and seed yield/plant using artificial neural networks (ANNs)

**Figure 11:**
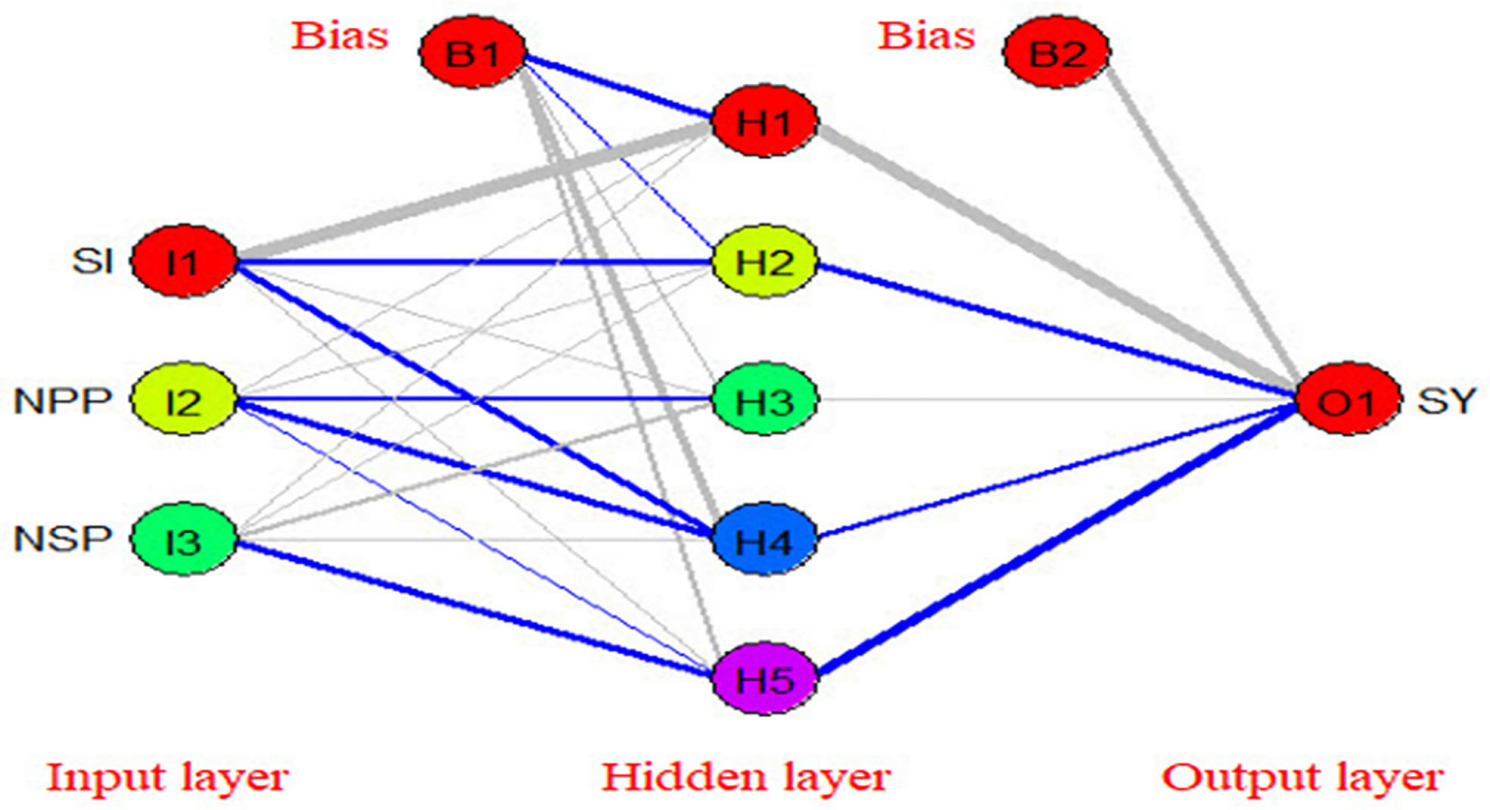
The structure of the used artificial neural network in the present study (blue color represents positive effect while grey color represents negative effect).

**Figure 12:**
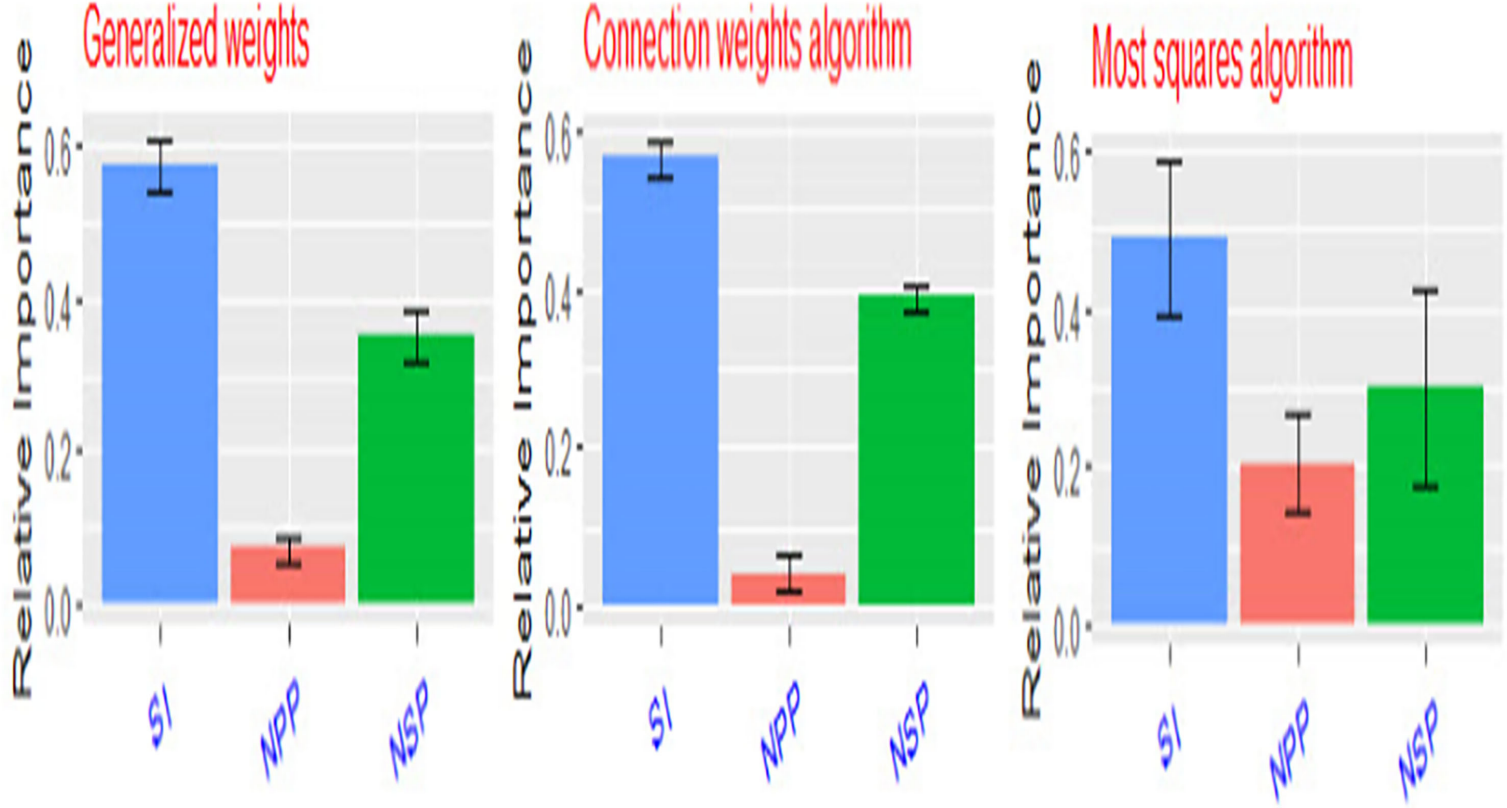
Relative importance of seed yield components (SI, weight of 100 seeds – NPP, number of pods/plant – NSP, number of seeds/pod) to seed yield/plant using three different methods.

Artificial neural networks (ANNs) are robust mathematical models. They consist of numerous and simple processing interconnected units known as neurons with a structure that is similar to the biological neurons in human brains (Zhongheng, 2016). Neurons in ANNs are arranged in layers such as the input layer, hidden layer, and output layer (**Figure 11**). The neurons in one layer are connected to the neurons in the next layer, but not to the neurons in the same layer. The strength of the connection weights among two neurons between the layers is used to estimate the relative importance of the inputs to the output and can be expressed as a fraction or %. In the present experiment, a three-layer feed-forward multilayer perceptron neural network (MLP) was used using a back propagation algorithm to model the relationship between yield components and seed yield/plant. The relative importance of yield components to seed yield/plant using three different methods **Figure 12** (Ibrahim et al., 2022). The seed index or weight of 100 seeds was the most important yield component to seed yield followed by the number of seeds per pod (NSP), while the number of pods/plant was the least important yield component to seed yield/plant.

## Discussion

### Si alleviated Cd-induced pea growth and yield reduction

Cd toxicity significantly reduced the growth characteristics of pea plants including plant height, leaf area, and shoot, root dry weight. Indeed, the inhibitory effects varied according to plant age, duration of exposure, heavy metal concentration, and type of heavy metal **(** Sytar et al., 2019; Ahmad et al., 2020). In line with our findings, Cd also reduced the growth of other plant species (EL-Okkiah, 2015; Perveen et al., 2016; Sun et al., 2016; Chen et al., 2019). In this regard, Cd stress inhibits root and stem cell growth by inhibiting cell elongation and cell division *via* an inhibition activity of the glycolysis pathway (Dalla Vecchia et al., 2005). Cd toxicity minimizes smitotic division of meristematic cells, which leads to reduced root length and dry biomass, and enhanced root diameter (Seth et al., 2008; Gratao et al., 2009; Abbas et al., 2017). Cd exposure also negatively affects multifactorial levels such as decreased nutrient uptake, photosynthetic efficiency, amino acids, protein metabolism, and water relations (Haider et al., 2021). In many agronomic crops such as pea, maize, barley (Popova et al., 2012), mungbean (Wahid et al., 2008), and wheat (Abbas et al., 2017), short- and long-term exposure to Cd toxicity inhibits photosynthetic activity. In this context, a 35.9% reduction in photosynthetic pigments was achieved by (Mayonde et al., 2021).

On the other hand, Si concentration in plants positively correlated with plant growth. Hussain et al., 2019 considered Si to be a growth regulator and played a defensive role against various biotic and abiotic stresses in previous research. Furthermore, Si application may have contributed to improved pea growth performance under stress and non-stress conditions by increasing photosynthesis as indicated by increased chlorophyll levels (Galal et al., 2021). The photosynthetic pigments (Chl a and Chl b) were significantly reduced by Cd toxicity. Cd stress causes a decrease in chlorophyll content (Gaballah and; Rady, 2012 and EL-Okkiah, 2015). Chlorophyll value is diminished in cadmium-treated plants due to inhibition of its biosynthesis as well as Cd-induced lower Mg and Fe concentrations (Gill et al., 2013). It is known that Si increases cellulose, hemicellulose, and lignin contents in rice cell walls in rice plants (Zhang et al., 2015). Higher cellulose and hemicellulose depositions due to Si result in forming a cell wall and consequentially inhibit heavy metal absorption (Zhang et al., 2020). Sugars are the most important soluble constituent that assists plants with osmotic adjustment. Sugars also provide energy to rapidly growing cells as well as the carbon skeletons needed to synthesize organic compounds (Taiz and Zeiger, 2010).

### Si treatment mitigates oxidative damage in Cd stressed plants

It is well known that heavy metal including Cd stress causes oxidative damage to plants, which can be exacerbated both directly and indirectly by increasing cellular ROS concentrations and decreasing cellular antioxidant capacity (Livingstone, 2001). Here we also reported increased lipid peroxidation (MDA), consistently, high MDA levels in pea shoots from Cd-treated plants were recorded (Ahmad et al., 2016; Chen et al., 2018). The observed increases were correlated with increased H_2_O_2_ levels. In this regard, Cd toxicity caused cell death in tobacco cells due to the accumulation of NADPH-oxidase and the over-production of H_2_O_2_ in fatty acids (Gill and Tuteja, 2010). Cadmium toxicity may stimulate ROS development in the mitochondrial electron transfer chain (Heyno et al., 2008).

Regardless of the fact that Cd is not an oxidative metal and cannot produce ROS directly through Fenton and Haber-Weiss reactions, indirect ROS production is common in Cd-exposed plants (Shahid et al., 2016). ROS can react with proteins, lipids, and nucleic acids at high concentrations, causing structural and functional changes including lipid peroxidation, membrane leakage, DNA breakage, and mutation (Apel and Hirt, 2004; Andresen and Küpper, 2013). They have the ability to oxidize proteins, fatty acids, and nucleic acids, which frequently results in cell structure changes and mutagenesis (Younis et al., 2016). Cd stress also results in damage to plant membranes and the destruction of cell biomolecules and organelles (Abbas et al., 2017).

To cope with Cd-induced oxidative stress, Pea plants increased the activities of antioxidant enzymes like POD and CAT. Interestingly, foliar treatment with Si further increased these activities under Cd stress conditions. Similarly, Cd-induced antioxidant enzyme activation, possibly through gene expression modulation or Cd-induced inhibition of enzyme inhibitors (Rahman et al., 2017). Another mechanism by which Si alleviated Cd stress in plants was stimulating antioxidant systems in plants (Shi et al., 2005). These antioxidants worked together to alleviate oxidative injury in Si treatment (Shen et al., 2010).

Therefore, Cd may indirectly contribute to the production of ROS through disruption in the chloroplasts of leaves (Gallego et al., 2012).

### Cd stress affects pea primary metabolites

Cd accumulation in the root was much greater than in the shoot. This finding is consistent with previous research on various plant species (Gaballah and Rady, 2012; Mostofa et al., 2015; Ahmad et al., 2016). Because Cd accumulated in the root is incapacitated by the cell wall and extracellular carbohydrates, higher Cd content in the root as compared to the shoot is one of the most important defense mechanisms of plants against Cd toxicity (Rahman et al., 2017). (Ahmad et al., 2016). Si application may reduce Cd accumulation in shoots; this indicates that silicon significantly negatively impacted Cd transport from roots to shoots, limiting it to root tissues. Similar findings have been reported in other monocotyledon species, including rice (Shi et al., 2005 and Zhang and Ge 2008). Kastori et al. (1992) discovered that the content of soluble proteins in *Helianthus annus* decreased with increasing heavy metal concentration. Protein content may be affected by heavy metals due to: (i) increased protein hydrolysis, resulting in a lower concentration of soluble proteins (ii) Lead’s catalytic activity **(** Bhattacharyya and Choudhuri, 1997); (iii) Protein synthesis decreasing under all stress conditions. (El-Beltagi et al., 2010; Ibrahim et al., 2012). Sugar levels in pea shoots were significantly higher in Si-treated plants, but not under Cd stress (50 mg/Kg soil).

### Cd stress altered the anatomical structure of pea organs but to less extend under Si treatment

Heavy metal can induce alterations in anatomical parameters. Furthermore, changes in anatomical traits were rather highly reliant on Cd concentration, with the changes becoming more noticeable as Cd concentration increased. Thus, the current study found that increasing concentrations of Cd had a negative impact on the anatomical structures of the pea, despite the plant’s preventing and mitigating such effects by modifying cellular structures to some extent. Among anatomical changes, degraded and smaller mesophyll tissue, the disintegration of parenchymatous tissues, cortical tissue loosening in roots of *Phaseolus vulgaris* L. (Talukdar, 2013), shriveling and cell breakdown, which resulted in cortical cell shape loss in *Phaseolus aureus* (Singh and Agrawal 2007) and reduced cortical thickness in maize roots (Gowayed and Almaghrabi, 2013). Similarly, Cd accumulation in *Pteris vittata* caused root cortical and endodermal cell breakdown or reduction (Sridhar et al., 2007; Armendariz et al., 2016). Heavy metals can affect the balance of root hormones, affecting cellular tissue differentiation and the number of cells in these tissues (Sandalio et al., 2001). Furthermore, the increased number of tracheary elements and decreased metaxylem area alter water storage capacity. Larger diameter vessels are more productive but less safe, leading to an increased risk of blisters (Dickison, 2000).

Moreover, *Thalassia hemprichii* exposed to Pb had wider cortical air spaces, and rising Pb concentration increased exodermal and endodermal cell wall thickness (Tupan and Azrianingsih, 2016). Cu promoted endodermal cell wall thickening in the bean (Bouazizi et al., 2010) and exposure separated the vascular bundles and pith regions (Talukdar, 2013).

Even though xylem conduits are tight and less susceptible to damage due to lignification, breakdown, loss of shape, or change in diameter have been observed in Cicer arietinum roots after Cr stress (Mondal et al., 2013). Lignin deposition in cortical cell walls can aid in the repairs of root turgescence by reducing water loss from the root *via* refluxas an adaptive mechanism that aids in root architecture stability (Hose et al., 2001).

Here, Cd strongly induced stomatal closure and decreased stomatal conductance in pea leaves. In line with these findings, several studies have found that Cd strongly induces stomatal closure in different plant species (Sun et al., 2016; Sadeghipour, 2018). Stomata closed individually under Cd stress, regardless of water status. Furthermore, Cd caused stomatal closure as a result of Cd entry into the guard cells, and it reduced the number of stomata per unit area (Ismael et al., 2019). Cd toxicity reduced stomatal conductance and cell division (Abbas et al., 2017). The following mechanisms of Si inhibition of metal transport in plants have been proposed: (1) thickening Casparian strips (2) depositing lignin in the cell walls and (3) depositing Si in the cell (Shi et al., 2005; Da Cunha and Do Nascimento, 2009).

## Conclusions

Cd has a toxic effect on pea plants, according to our research. Cd toxicity altered root and stem growth, anatomy, and biochemistry. Plants exposed to Cd toxicity showed reduced growth, which was correlated with Cd accumulation and reduced chlorophyll and primary metabolites such as sugars and protein. Additionally, Cd significantly oxidative damage (MDA and H_2_O_2_), and to cope with stress plants increased their enzyme activity (CAT, POD) compared to controls. Higher Cd concentrations appear to cause anatomical abnormalities such as a distinct vascular system, abnormal lignification in the pith parenchyma, and enlarged cortical cells, according to our findings. The use of Si effectively reduced the oxidative burst caused by Cd toxicity. Therefore, we recommend using Si at 300 ppm as a promising approach to reduce Cd toxicity

## Data availability statement

The original contributions presented in the study are included in the article/supplementary material. Further inquiries can be directed to the corresponding author.

## Author contributions

All authors have contributed equally to the research and analysis of the various results sections within the review. All have corrected and modified the different versions of the manuscript as prepared by the corresponding and senior authors. All authors read and approved the final manuscript.

## Funding

Princess Nourah bint Abdulrahman University Researchers Supporting Project number (PNURSP2022R214), Princess Nourah bint Abdulrahman University, Riyadh, Saudi Arabia.

## Acknowledgments

The authors are grateful to the Researchers Supporting Project number (PNURSP2022R214), Princess Nourah bint Abdulrahman University, Riyadh, Saudi Arabia.

## Conflict of interest

The authors declare that the research was conducted in the absence of any commercial or financial relationships that could be construed as a potential conflict of interest.

## Publisher’s note

All claims expressed in this article are solely those of the authors and do not necessarily represent those of their affiliated organizations, or those of the publisher, the editors and the reviewers. Any product that may be evaluated in this article, or claim that may be made by its manufacturer, is not guaranteed or endorsed by the publisher.
